# Mental health services in Gauteng, South Africa: A proxy evaluation using pharmaceutical data

**DOI:** 10.4102/sajpsychiatry.v30i0.2157

**Published:** 2024-03-31

**Authors:** Lesley J. Robertson, Jade C. Bouwer

**Affiliations:** 1 Department of Psychiatry, Faculty of Health Sciences, University of the Witwatersrand, Johannesburg, South Africa; 2 District Specialist Mental Health Team, Sedibeng District Health Services, Vereeniging, South Africa; 3 Department of Psychiatry, Faculty of Health Sciences, University of Cape Town, Cape Town, South Africa; 4 Valkenberg Psychiatric Hospital, Department of Health and Wellness, Cape Town, South Africa

**Keywords:** mental health systems, access to mental and neurological healthcare, public mental health, pharmacoepidemiology, ATC and DDD system, essential medicines, South Africa

## Abstract

**Background:**

South African legislation advocates for equitable access to mental healthcare services integrated into general healthcare settings. Mental, neurological, and substance use (MNS) disorders are often comorbid. Pharmacoepidemiology provides indirect evidence of service provision for conditions amenable to medicine treatment.

**Aim:**

The study aims to evaluate medicine procurement for MNS disorders at different service levels in the health system.

**Setting:**

The Public health sector, Gauteng province formed the setting for the study.

**Method:**

A secondary analysis of the Gauteng pharmaceutical database was conducted using Anatomic Therapeutic Chemical (ATC) and defined daily dose (DDD) methodology. Anatomic Therapeutic Chemical classes of medicines for MNS disorders were included. Defined daily doses and costs were calculated per 1000 population served by each facility and service level. Statistical comparisons were made using chi-square testing.

**Results:**

General healthcare settings accounted for 90% (R118 638 248) and specialised hospitals for 10% (R13 685 032) of expenditure on medicines for MNS disorders, procuring 94% (*n* = 49 442 474) and 6% (*n* = 3 311 528) of DDDs, respectively. Although district clinics procured 60% of DDDs, they procured the least per 1000 population served, whereas district hospitals procured the most. For almost all ATC classes, procurement differed significantly between municipalities at every service level and between specialised hospitals.

**Conclusion:**

In Gauteng province, most medicines for MNS disorders are procured by general healthcare services, but access to care may not be equitable. While population coverage at district clinics appears low, district hospitals may experience the greatest care burden. Research regarding quality of care at each service level is recommended.

**Contribution:**

This study provides insight into service provision for MNS disorders.

## Introduction

Promulgated in 2004, the *South African Mental Health Care Act No.17* of 2002^1^ advocated for mental healthcare services to be made available ‘equitably, efficiently, and in the best interests of mental health care users within the limits of the available resources’ (Section 3[a][i]); integrated into the general healthcare setting (Section 3[a][iii]); and provided at primary, secondary, and tertiary levels of healthcare (Section 4[a]). These objectives were a stark contrast to the custodial care psychiatric hospitals already established in South Africa and extensive reorganisation of the mental healthcare system was needed to meet them.

Almost a decade later, the National Mental Health Policy Framework and Strategic Plan 2013–2020 (NMHPF) outlined a 12-point action plan to address persistent inadequate and inequitable mental healthcare services in South Africa.^2^ The same 12 areas for action are detailed in the updated National Mental Health Policy Framework and Strategic Plan 2023–2030.^3^ These actions are as follows: (1) services should be organised to align with World Health Organization (WHO) recommendations, (2) the mental healthcare system should be monitored and evaluated, and (3) psychotropic medicines would be available at all service levels in accordance with the standard treatment guidelines and essential medicines list.

### Organisation of mental healthcare services

**FIGURE 1:**
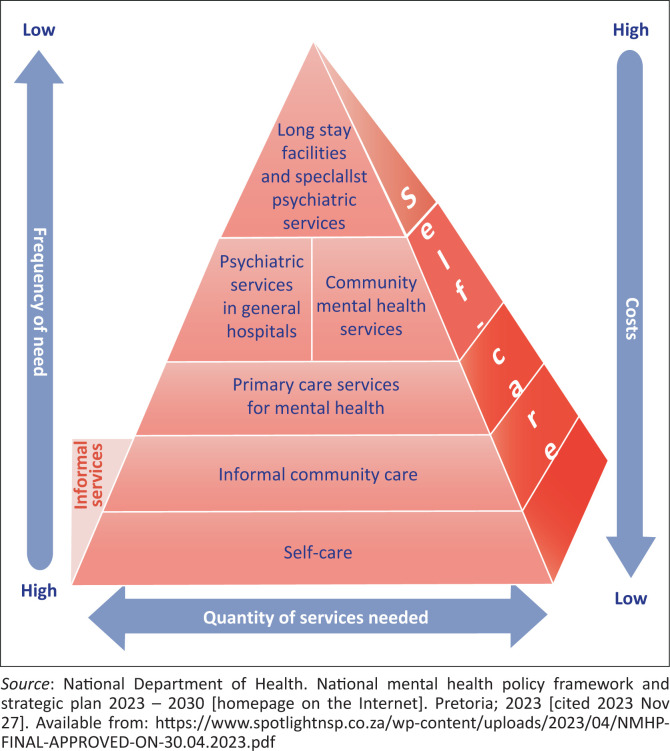
World Health Organization of services for mental healthcare.

In its organisation of services for optimal mental healthcare (Figure 1), the WHO recommends that maximum care is provided in the general healthcare setting rather than specialised hospitals.^3^ While primary healthcare (PHC) is the first point of care, specialist care should be provided in general hospitals or as part of community mental health services for people with complex or severe conditions. Only those whose mental disorders are too severe for PHC, community mental health services, and general hospital psychiatry are referred to specialised psychiatric or long-stay hospitals. Thus, equitable access to care is assured through PHC, stigma is reduced through service delivery in general health settings, and costs contained by limiting institution-based care.

### Monitoring and evaluation

The NMHPF recommended that mental healthcare indicators are integrated into the district health information system (DHIS) to facilitate routine monitoring, evaluation, and planning of mental health services.^2^ While several mental health indicators have been integrated into the DHIS,^4^ evaluation of health systems is complex, requiring research and analysis from multiple perspectives. Pharmacoepidemiology may provide insight into a health system, at least regarding services for people with illnesses amenable to medicine treatment, as it yields indirect information regarding service usage and burden of disease.^5^ To facilitate medicine utilisation research, the WHO Collaborating Centre for Drug Statistics Methodology and the Norwegian Institute of Public Health developed the Anatomical Therapeutic Chemical (ATC) and Defined Daily Dose (DDD) classification system.^6^

In the ATC and DDD system, a medicine’s ATC code has five levels, denoting its main anatomical group, its therapeutic, pharmacological, and chemical subgroups and its chemical substance. For example, in the ATC code for haloperidol (N05AD01), N stands for nervous system, 05 for psycholeptics, A for antipsychotics, D for butyrophenone derivatives, and 01 for haloperidol. The DDD is a defined daily dose assigned to each medicine according to its ATC code (for haloperidol, the DDD is 8 mg whether given orally or parenterally). The DDD is not a clinical recommendation but is a standardised unit of measurement. Thus, when DDDs are calculated according to population size (e.g. per 1000 inhabitants or per 100 bed days) the ATC and DDD system enables comparison of medicine utilisation between healthcare regions, settings, or facilities.

### Mental healthcare services in Gauteng

The organisation of mental healthcare services in Gauteng province, as described by the provincial mental health directorate,^7^ are presented in Table 1. The levels of care and corresponding service levels differ slightly from those of the WHO. Consistent with national healthcare referral policy guidelines,^8^ mental healthcare through PHC services includes outpatient care at district clinics and acute inpatient care at district hospitals and is provided by non-specialist medical practitioners.

**TABLE 1:** Gauteng provincial mental health services and levels of care.

WHO mental health service level	Gauteng province levels of care	Gauteng province service levels
Mental health through primary care services	Primary mental healthcare	District clinics
	Acute non-specialist inpatient care	District hospitals
Specialist mental health services in general health settings	Community mental healthcare	District clinics
	Psychiatric care in general hospitals	Regional hospitals
		Tertiary hospitals
		Central academic hospitals
Specialised services in stand-alone institutions	Psychiatric hospital care	Psychiatric hospitals
	Long-stay hospital care	Rehabilitation centres and privately contracted hospitals

WHO, World Health Organization.

Furthermore, the community mental health services are a specialist mental healthcare service delivered from district clinics, alongside PHC. They do not have a designated service level, as they are not in national referral policy guidelines,^8^ planning and spending,^9^ or monitoring and evaluation.^4,10^ The community mental healthcare services were initiated in Gauteng province in the early 1990s, as deinstitutionalisation commenced.^11,12^ However, they are indistinguishable from district clinic services in provincial healthcare monitoring, including the monitoring of pharmaceutical procurement and expenditure.

Although deinstitutionalisation progressed rapidly in Gauteng province, there is little information regarding service utilisation for mental, neurological and substance use (MNS) disorders in the health system. The lack of data was made acutely apparent in 2016 and 2017, during the ‘Life Esidimeni tragedy’, which also exposed poor preparation in the general healthcare setting for deinstitutionalised people with severe mental illness.^13^ Without adequate monitoring, it is difficult to understand the access to or burden of care for MNS disorders at different service levels. However, medicine procurement data are available in Gauteng province, and may provide insight into service utilisation for people with conditions amenable to medicine treatment.

This study aimed to gain insight into the health services for MNS disorders in Gauteng province using medicine procurement data. The objectives were to analyse the procurement of and expenditure on medicines for MNS disorders in the Gauteng Provincial public health sector for the 2017/2018 financial year, in proportion to total medicine expenditure, the population served, and in relation to the different service levels. The study was conducted in partial fulfilment of a master’s in medicine degree.^14^ The costs and DDDs of each medicine procured are reported in Bouwer et al.,^15^ the first publication from the study. In this article, the evaluation of medicines procured for MNS disorders in proportion to the population served at each service level is presented.

Based on South African legislation and policy as well as the history of deinstitutionalisation in the province, we hypothesised that:

- most medicines for MNS disorders would be procured for use in the general healthcare setting.
- there would be no difference in procurement between the different municipalities of Gauteng province for each service level in the general healthcare setting, reflecting equitable access to medicines across the province.
- all medicine classes would be procured at all service levels, reflecting access to care at primary, secondary, and tertiary services as well as specialised hospitals, according to the standard treatment guidelines and essential medicine list.
- in proportion to the population served at each service level, the greatest procurement of medicines would be at the district clinic service level (including PHC and community mental health services).

## Methods

A secondary analysis of the Gauteng Department of Health pharmaceutical database was conducted using the ATC and DDD methodology. A brief outline of the methods is provided here; the reader is referred to Bouwer et al.^15^ for a more detailed explanation.

### Study setting

The study was set in Gauteng province public health sector. The province has five constituent municipalities: three metropolitan municipalities (City of Johannesburg, Ekurhuleni, and Tshwane) and two district municipalities (Sedibeng and West Rand). The healthcare facilities in each municipality are presented in Table 2.

**TABLE 2:** Service levels and healthcare facilities in each municipality.

Municipality	Service level	Healthcare facility
City of johannesburg Metropolitan municipality	District clinic†	COJ regional pharmacy
	District hospital	Bheki mlangeni hospital South rand hospital
	Regional hospital	Edenvale hospital Rahima moosa mother and child hospital Helen joseph hospital
	Central hospital	Charlotte maxeke johannesburg academic hospital Chris hani baragwanath academic hospital Tara h. Moross centre (psychiatric) Sizwe hospital (multidrug resistant tuberculosis)
Ekurhuleni metropolitan municipality	District clinic†	Ekurhuleni regional pharmacy
	District hospital	Bertha gxowa hospital
	Regional hospital	Far east rand hospital Pholosong hospital Tambo memorial hospital Thelle mogoerane hospital
	Tertiary hospital	Tembisa hospital
Sedibeng district municipality	District clinic†	Sedibeng regional pharmacy Kopanong hospital Heidelberg hospital
	Regional hospital	Sebokeng hospital
Tshwane metropolitan municipality	District clinic†	Tshwane regional pharmacy Tshwane district hospital Pretoria west hospital Jubilee hospital Odi hospital Bronkhorstspruit hospital
	Regional hospital	Mamelodi hospital
	Tertiary hospital	Kalafong hospital
	Central hospital	Dr george mukhari academic hospital Steve biko academic hospital
	Specialised hospital	Weskoppies hospital (psychiatric) Tshwane rehabilitation centre (physical disabilities) Cullinan care centre hospital (intellectual disabilities)
West rand district municipality	District clinic†	West rand regional pharmacy
	District hospital	Carletonville hospital Dr yusuf dadoo hospital
	Regional hospital	Leratong hospital
	Specialised hospital	Sterkfontein hospital (psychiatric)

*Source*: Bouwer JC. Procurement and expenditure of medicines used for mental, neurological and substance use disorders: A secondary analysis of the 2017/18 Gauteng pharmaceutical database (MMed Research Report) [homepage on the Internet]. Johannesburg: University of the Witwatersrand; 2020 [cited 2023 Nov 27]. Available from: https://wiredspace.wits.ac.za/server/api/core/bitstreams/f16a41fd-40c1-45f3-bf5c-1a73f301f608/content

† , Includes both primary mental healthcare and community mental health services.

General healthcare facilities serve the general population of their respective municipalities, with up-referral from district to regional (secondary), tertiary, or central (quaternary) hospitals as needed.^8^ The specialised hospitals take referrals from other healthcare facilities according to the nature of the person’s illness or disability rather than a demarcated catchment area.

### Study population

The study population comprised medicines procured for the management of MNS disorders in general and specialised healthcare settings of the public health sector. Medicines procured for privately contracted long-stay psychiatric hospital care were excluded.

Medicines from ATC classes N03A (antiepileptics), N04A (anticholinergics), N04B (dopaminergics), N05A (antipsychotics), N05B (anxiolytics), N05C (hypnotics and sedatives), N06A (antidepressants), N06B (psychostimulants and lithium), and N07B (medicines used in addiction disorders) were included. One medicine in class N06B (caffeine citrate injection) that is not used in the treatment of MNS disorders was excluded from this analysis, although it was captured in the data-collection process.^15^

### Data collection

The quantities and costs of each medicine and medicine formulation procured during the 2017/2018 financial year were collected from the Gauteng Medical Supplies Administration System database. The population served by each healthcare facility in each municipality was obtained from the DHIS, which records headcounts attending district clinics and patient day equivalents at hospitals.

### Data analysis

Expenditure on and number of DDDs of each medicine procured were calculated and expressed as cost/1000 population served and DDD/1000 population served, respectively. Statistical analyses were conducted using R software.^16^ Tests were two-tailed probability values, with statistical significance accepted when α ≤ 0.05. Chi-squared (χ^2^) contingency tables were used to compare procurement of medicines between municipalities at each service level in DDD/1000 and the cost/1000 population served.

### Ethical considerations

Ethics approval was obtained from the Human Research Ethics Committee at the University of the Witwatersrand (ethics approval no. M180612). Permission to analyse and publish the data was granted by the Gauteng Department of Health Research and Epidemiology Directorate.

## Results

During the 2017/2018 financial year, just over 130 million rand was spent on medicines for MNS disorders with the procurement of almost 52.8 million DDDs for use in the public health sector (Table 3). Procurement for use in general healthcare settings accounted for 90% (R116 542 669) of expenditure on medicines for MNS disorders and 94% (*n* = 49 442 317 DDDs) of DDDs. The largest proportion of DDDs (60%) was procured for use at district clinics, by PHC and/or community mental health services. Medicines procured for hospital use were more expensive than those procured for district clinics, with the costliest procured by specialised hospitals.

**TABLE 3:** Costs and defined daily doses procured at each service level.

Service level	Cost		DDDs procured		Cost/ DDD
	SA Rand	%	*N*	%	
District clinic	R60 027 104.61	46.0	31 430 245	60.0	R1.91
District hospital	R8 593 349.27	7.0	4 241 494	8.0	R2.05
Regional hospital	R11 423 385.04	9.0	3 970 055	8.0	R2.94
Tertiary and central hospital	R36 498 829.30	28.0	9 800 523	19.0	R3.90
Specialised hospital	R13 685 032.84	10.0	3 311 528	6.0	R4.13
Total for gauteng province	R130 227 701.06	100.0	52 753 845	100.0	R2.51

DDDs, defined daily doses; R, Rand; SA, South African.

### Medicine procurement in general healthcare settings

The cost and number of DDDs for each service level in general healthcare facilities of each municipality are presented in Table 4. There was no significant difference between municipalities in the population served by their respective health systems relative to their general populations, and no difference between municipalities in the percentage of the population served at district clinics and district hospitals. However, municipalities differed in the proportion of population served at regional, tertiary, and central hospitals.

**TABLE 4:** Costs and defined daily doses of medicine procured in general healthcare settings per municipality.

Item	City of Johannesburg	Ekurhuleni	Sedibeng	Tshwane	West rand	χ^2^	*df*	*p*
Total population (2011 Census)^17^	4 434 827.00	3 178 470.00	916 484.00	2 921 488.00	820 995.00	-	-	-
Population served (*n*)	10 049 491.00	7 148 680.00	2 161 353.00	6 255 309.00	2 195 153.00	-	-	-
Population served: total population (*n*)	2.27	2.25	2.36	2.14	2.67	0.07	4	0.999
Population served at district clinics (%)	75.00	80.00	80.00	70.00	78.00	0.93	4	0.920
Population served at district hospitals (%)	2.00	2.00	6.00	7.00	6.00	5.04	4	0.283
Population served at regional hospitals (%)	2.00	13.00	14.00	2.00	16.00	19.91	4	0.001
Population served at tertiary and central hospitals (%)	21.00	5.00	NA	21.00	NA	10.89	2	0.004
Total cost	R46 751 036.12	R21 687 469.05	R9 958 050.96	R30 283 596.53	R7 862 515.56	-	-	-
Cost to district clinics (%)	47.00	62.00	75.00	40.00	61.00	13.23	4	0.010
Cost to district hospitals (%)	4.00	3.00	14.00	10.00	20.00	19.69	4	0.001
Cost to regional hospitals (%)	4.00	28.00	11.00	5.00	19.00	30.54	4	< 0.001
Cost to tertiary and central hospitals (%)	45.00	7.00	NA	45.00	NA	29.77	2	< 0.001
Total DDDs procured (*n*)	18 816 587.00	10 656 631.00	5 077 019.00	11 132 854.00	3 759 227.00	-	-	-
DDDs procured by district clinics (%)	62.00	73.00	80.00	52.00	60.00	7.51	4	0.111
DDDs procured by district hospitals (%)	3.00	3.00	13.00	15.00	25.00	28.88	4	< 0.001
DDDs procured by regional hospitals (%)	3.00	20.00	7.00	4.00	15.00	22.33	4	< 0.001
DDDs procured by tertiary and central hospitals (%)	32.00	4.00	NA	29.00	NA	21.82	2	< 0.001
Total cost/1000 population served	R4652.08	R3033.77	R4607.32	R4841.26	R3581.76	605.25	4	< 0.001
Total DDDs/1000 population served	1872.39	1490.71	2349.00	1779.74	1712.51	203.65	4	< 0.001
Cost/DDD	R2.48	R2.04	R1.96	R2.72	R2.09	0.19	4	0.996

DDD, defined daily dose; NA, not applicable; R, South African Rand; *df*, degrees of freedom.

The expenditure on and procurement of medicines for MNS disorders differed between municipalities at each service level, except for the DDDs procured by district clinics. The difference in expenditure but not in DDDs at district clinic level suggests similar doses of different types of medicines at varying expense.

The cost/DDD procured did not differ significantly between municipalities. However, when multiplied by the number of DDDs procured, the differences in cost/1000 population served are significant. Thus, in Tshwane, the highest cost/1000 population served is linked to the procurement of more expensive medicines, possibly related to Tshwane having the greatest proportion of people treated in hospitals rather than district clinics compared with the other municipalities. However, in Ekurhuleni, the lower cost/1000 population served is related to the procurement of fewer DDDs/1000 population served as well as less expensive medicines than Tshwane.

### Medicine procurement for specialised hospitals

The specialised hospitals serve the entire Gauteng population, taking referrals from general healthcare facilities. They are separated into two groups: rehabilitation centres and psychiatric hospitals (Table 5). Being specialised, both groups served a very small number of people. While the high cost/1000 population served at psychiatric hospitals is related to the entire patient population being treated for MNS disorders, the high cost/DDD means that the use of more expensive medicines is a contributing factor.

**TABLE 5:** Cost and defined daily doses of medicine procured at specialised hospitals.

Item	Rehabilitation centres	Psychiatric hospitals
Total Gauteng population (Census 2011) (*n*)	12 272 264.00	12 272 264.00
Population served (*n*)	131 999.00	504 838.00
Population served: total population (*n*)	0.01	0.04
Total cost	R395 813.00	R13 289 220.00
Total DDDs procured	179 173.00	3 132 355.00
Cost/1000 population served	R2998.61	R26 323.73
DDDs/1000 population served	1357.38	6204.67
Cost/ DDD	R2.21	R4.24

DDDs, defined daily doses; R, South African Rand.

### Anatomic Therapeutic Chemical classes procured per 1000 population served at each service level

**FIGURE 2:**
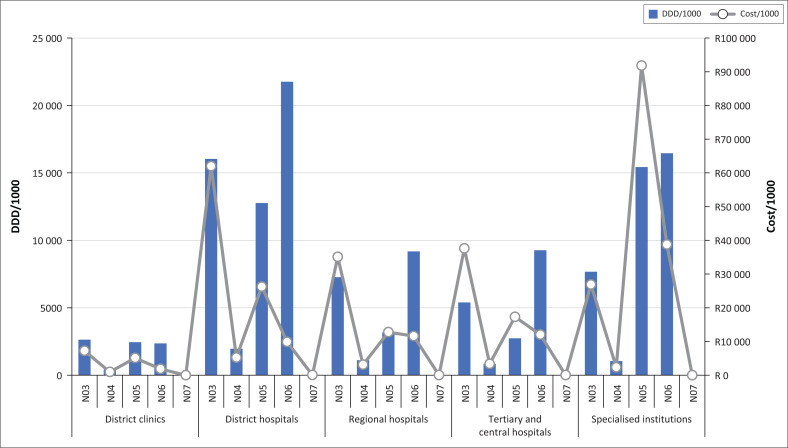
Anatomic Therapeutic Chemical classes procured per thousand population served at each service level.

The costs and DDDs procured for the different ATC classes at each service level are illustrated in Figure 2. For simplicity, ATC subclasses N04A and N04B (anticholinergics and dopaminergics, respectively); N05A, N05B, and N05C (antipsychotics, anxiolytics, and hypnotics and sedatives, respectively); and N06A and N06B (antidepressants and psychostimulants, respectively) were combined in their particular classes.

All medicine classes were procured for use at all service levels except for class N07B (medicines for addiction disorders). Only one medicine (methadone syrup) in class N07B was procured by the province, and it was procured in small quantities at all general hospital service levels but not at district clinics or specialised hospitals.

Per population served in the general healthcare setting, district clinics procured the least and district hospitals the largest quantity of DDDs. This finding suggests that, even though district clinics procured 60% of all DDDs, most people attending this level of care are not prescribed medicines for MNS disorders. By contrast, district hospitals appear to carry the greatest care burden in proportion to their size.

Cost-drivers at tertiary and central hospitals came from ATC classes N03 (antiepileptics) and N05 (antipsychotic, anxiolytic, and hypnotic medicines). At specialised hospitals, the most expensive medicines came from class N05:

- /1000 = per thousand population served; DDD = defined daily dose;
- N03 = N03A (antiepileptics); N04 = N04A (anticholinergics) and N04B (dopaminergics);
- N05 = N05A (antipsychotics), N05B (anxiolytics), and N05C (hypnotics and sedatives);
- N06 = N06A (antidepressants) and N06B (psychostimulants);
- N07 = N07B (medicines used in addiction disorders)

### Comparisons in Anatomic Therapeutic Chemical class procurement between facilities at each service level

The procurement of ATC classes N03A (antiepileptics), N05A (antipsychotics and lithium), N05B (anxiolytics), N06A (antidepressants), and N06B (psychostimulants) per 1000 population was analysed for each municipality (and health facility at tertiary, central, and specialised hospitals) at each service level. The results of these comparisons are available in the appendix.

In the general health setting (Figures 1–A1, Figure 2–A1, Figure 3–A1, Figure 4–A1 and Figure5–A1 in Online Appendix 1), procurement differed significantly between municipalities for almost all ATC classes at every service level. Similar procurement of DDDs/1000 population served was found for anxiolytics at district clinic (*p* = 0.064) and tertiary hospital (*p* = 0.134) levels, and for psychostimulants at tertiary hospitals (*p* = 0.055). The cost/1000 population served differed for all ATC classes except for antidepressants at the district clinic level (*p* = 0.3). As the specialised hospitals (Figure 6 in the appendix) serve specific patient populations, differences in procurement are to be expected. However, the number of DDDs/1000 population served procured by one psychiatric hospital is noticeably greater than those procured by the other two psychiatric hospitals.

## Discussion

In this analysis of medicine procurement for the treatment of MNS disorders in Gauteng province, we found that most DDDs (94%) were procured for use in the general healthcare setting, consistent with our first hypothesis. We also found that medicines from each included ATC class were procured for use at every service level, except for medicines from ATC class N07B (medicines used in the treatment of addiction disorders). However, we did not find evidence to support equitable access to treatment of MNS disorders across the province. Furthermore, although district clinics procured the largest quantity of DDDs compared to other service levels, they procured the lowest number of DDDs in proportion to the population they served, rejecting our last hypothesis.

### Integration of care for mental, neurological and substance use disorders

Our finding that most medicines were procured for use in the general healthcare setting suggests that the care for people with MNS disorders has been integrated into general healthcare services in Gauteng province. Our study therefore supports the finding of integrated care by Docrat et al.^18^ in their analysis of government expenditure on the management of MNS disorders during 2016/2017.

For Gauteng province, Docrat et al.^18^ found that 75% of hospital admissions for MNS disorders were at general hospitals (26% at district, 22% at regional, and 27% at tertiary and central hospital levels) and 25% at specialised hospitals (22% at the three psychiatric hospitals and 3% at rehabilitation centres). In addition, 86% of outpatient care was at the district clinic level, 2% at general hospitals, and 12% at specialised hospitals (11.7% and 0.3% at the psychiatric hospitals and rehabilitation centres, respectively). Of concern is the fact that mental health expenditure did not reflect service usage. Docrat et al.^18^ report that the Gauteng Department of Health spent 6.2% of its total healthcare expenditure on public sector services for MNS disorders, of which 89% was on inpatient and 11% on outpatient care. Almost two thirds (65%) of expenditure went on specialised hospitals (49% at psychiatric hospitals and 16% at rehabilitation centres). Of the remaining expenditure, 18% was at tertiary and central hospitals, 4.5% at regional hospitals, 4% at district hospitals, and 8.5% at district clinics.

As Docrat et al. may not have captured all expenditure on MNS disorders in general healthcare settings, the expenditure on resources for the MNS disorders represented by the medicines included in our study might differ. Nevertheless, it does appear that resource distribution may not match demand for care at district clinics and general hospitals.

In particular, the quality of care for people with MNS disorders at district and regional hospital levels warrants further investigation. Importantly, psychiatric services are not included in the standard package of care for district hospitals and are optional at regional hospitals.^8^ However, in proportion to the population served at each level, district hospitals procure the most medicines for MNS disorders, and regional hospitals procure quantities similar to tertiary and central hospitals, which do include psychiatric services in their package of care.

### Equitable access to treatment of mental, neurological and substance use disorders

For all health conditions, the DHIS data suggest that there is equitable entry into the healthcare system at district clinic and district hospital level across Gauteng province. However, there is a difference between municipalities in the population served at regional, tertiary and central hospitals, indicating differences in reaching or obtaining care at more specialised service levels, possibly related to the geographical locations of these hospitals.

For MNS disorders, the medicine procurement data indicated availability of a range of medicines at every service level except for methadone (the only medicine procured from ATC class 70B in Gauteng province), which was only procured in small quantities by general hospitals. Non-procurement of methadone for district clinic use is consistent with the standard treatment guidelines, as opioid substitution therapy is not standard care in the South African public sector.^19,20^ Despite methadone being on the national essential medicine list, it is only used for the management of opioid withdrawal.

While there is procurement of medicines for mental and neurological disorders at all service levels, data differed between municipalities, suggesting that access to treatment for these conditions may not be equitable across the province, at any service level. However, medicine procurement data can only provide limited insight regarding access to healthcare.^5,21^ Levesque et al.^22^ conceptualise access to care as ‘the opportunity to have healthcare needs fulfilled’ with supply (health system related) and demand (user related) dimensions.

Although differences in availability of the various hospital services influence access to care in the different municipalities, other issues, and the associated healthcare outcomes, should be explored. Possible factors regarding healthcare supply include disparities in approachability and acceptability of the healthcare systems for people with mental and neurological disorders between municipalities. Factors affecting the demand for treatment include variations in mental health literacy in the general population, stigma, and individual experience of health services received.

Healthcare outcomes are important in terms of access to care, rational medicine use, and health service costs. Having one’s ‘healthcare needs fulfilled’ means that the treatment provided makes a positive difference to the individual.^22^ In chronic diseases, positive outcomes should improve the demand for and adherence to treatment. For people with MNS disorders, positive outcomes include improved psychosocial functioning and quality of life, as well as a reduction in symptoms and premature mortality.^23^ For a resource constrained country, greater medicine costs should be justified by improved outcomes.

### Primary mental healthcare and community mental health services

In its 2022 mental health report,^24^ the WHO no longer recommends specialised hospitals as part of the optimal mix of services for mental healthcare. Instead of a tiered system, the WHO now proposes a network of three groups of services:

- mental healthcare through general healthcare services (PHC and general hospitals)
- community mental health services
- mental healthcare through the non-health sector

The WHO emphasises that all countries, including low- and middle-income countries, should organise services in a manner, which provides community-based access to specialised care at district level for those with more severe mental disorders. While the WHO report is focussed on mental healthcare, the same principles are applicable to the care of people with neurological^25,26^ and substance use disorders.^27^

In our study, district clinics procured the greatest quantities of medicines for MNS disorders but appeared to treat the smallest proportion of the population served compared to the other service levels. How much medicine procurement was for use by primary care practitioners and how much by community mental health services is unknown. Nevertheless, there is no doubt that primary mental healthcare in South Africa requires strengthening.^28^ For better understanding of the system, additional factors to explore include the unmet need for medicine treatment of MNS disorders by primary care practitioners, the relative burden of care on community mental health services, and effectiveness of existing treatment approaches in optimising user well-being and preventing hospital admissions.

### Study’s limitations

There are several limitations to this study. Procurement data may not be a reliable proxy for medicine utilisation and service provision. Variations in supply chain management across the province and their influence on procurement are not considered. In addition, off-label prescribing could cause misinterpretation of the conditions being treated. The ATC classification does not always reflect psychopharmacological classifications (e.g. clonazepam is classed as an antiepileptic and lithium carbonate as an antipsychotic in the ATC system), which may cause some confusion in interpreting the findings. Nevertheless, the ATC and DDD system allows for a high-level analysis of a health system, enables identification of areas, which warrant more detailed medicine utilisation research, and facilitates the monitoring of changes over time.

## Conclusion

Notwithstanding the limitations, this study provides valuable insight into the healthcare services for people with MNS disorders in Gauteng province. Importantly, most medicine treatment of MNS disorders is in the general healthcare setting, access to treatment may not be equitable across the province, and medicine procurement at the district clinic level may be insufficient to meet population needs. Further research is recommended with regard to population coverage, rational medicine use, and the quality of care provided.
